# Total Knee Arthroplasty after Correction of Tibial Diaphyseal Nonunion with Clamshell Osteotomy

**DOI:** 10.1155/2018/2632963

**Published:** 2018-09-25

**Authors:** Pingal Desai, Vivek Sharma, Karanvir Prakash

**Affiliations:** ^1^Honorary Clinical Associate Professor of Orthopedics, University of Kansas, Lawrence, USA; ^2^Colonial Orthopedics, Colonial Heights, USA; ^3^Orthopedic Surgeon-Southwest Medical Center, Liberal, Kansas, USA

## Abstract

Total knee arthroplasty is mostly done to relieve pain and disability from a severe and degenerated knee. Deformities in the coronal and sagittal plane could be corrected with the help of cuts made in tibia and femur during total knee replacement as well as with ligament release. However, large deformities in the lower extremity particularly in the diaphysis region need correction prior to the total knee replacement. It helps to limit the amount of bone that will be cut and helps the ligament release. Several extra articular and intra-articular methods for the correction of diaphyseal deformity have been described. We present the case of clamshell osteotomy for the correction of diaphyseal deformity in the tibia and a total knee replacement after the osteotomy site healed.

## 1. Introduction

Clamshell osteotomy was first described by Russell et al. in 2009 to correct deformities in the long bone [1]. It involves two transverse osteotomies: one on the proximal and one on the distal aspect of malunion site of the long bone. The third longitudinal osteotomy is carried out in the fragment in between two transverse osteotomies. The osteotomized fragment would open like a clamshell and it would allow realignment of the mechanical axis of the extremity. This is followed by fixing proximal with distal fragment and stabilizing the osteotomy. We report the first case of total knee arthroplasty (TKA) after extra-articular correction of mechanical axis of lower extremity in tibia with clamshell osteotomy.

## 2. Case Report

**Figure 1:**
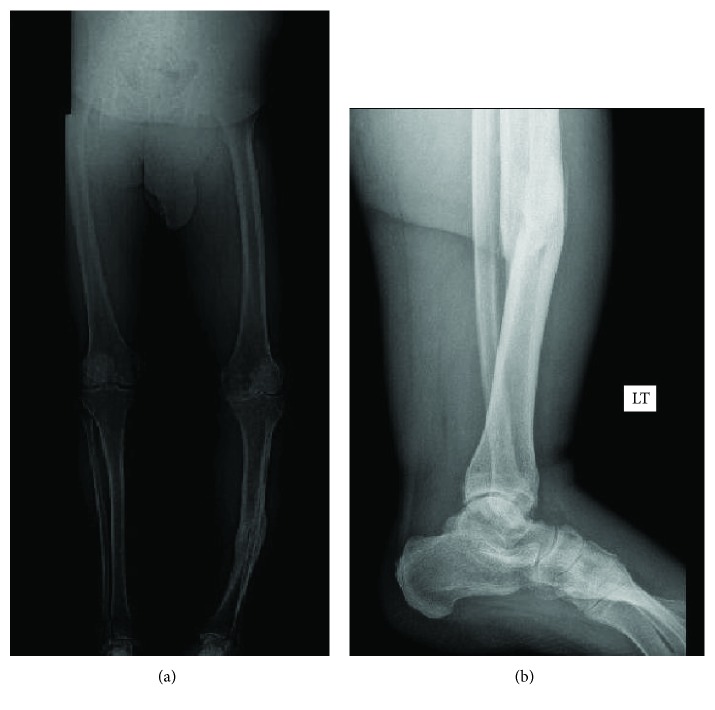
(a) Leg length view showing 30 degrees varus of tibia and several osteoarthritis left knee. (b) Lateral view showing 20 degrees apex anterior angulation at malunited fracture site.

**Figure 2:**
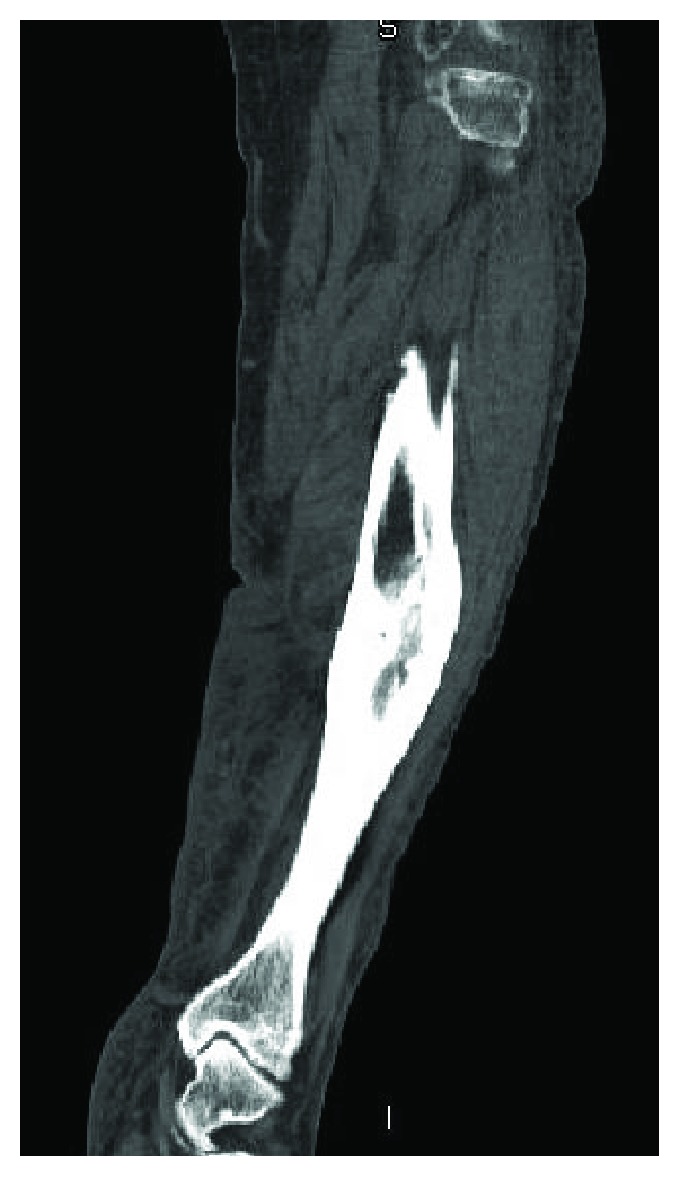
CT scan—malunited fracture of the middle third left tibia.

A 65-year-old male with a BMI of 42 and uncontrolled type II diabetes mellitus came to our clinic with a fracture on the left tibial shaft. He was treated conservatively for 20 years. Radiographs showed a malunited fracture on the middle third left tibia in 20-degrees varus, 15-degrees apex anterior angulation with a 1 cm anterior translation of distal segment, 20-degrees internal rotation and 1.5 cm shortening. He also had severe tricompartmental osteoarthritis on the left knee (Figures 1(a) and 1(b)). A CT scan confirmed the presence of malunion (Figure 2). Blood sugar was controlled by an endocrine physician and a clamshell osteotomy of the tibia was planned.

## 3. Surgical Procedure for Clamshell Osteotomy

**Figure 3:**
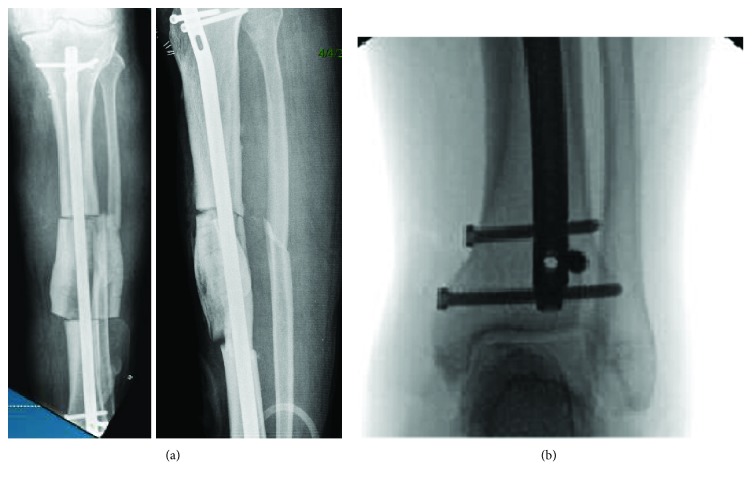
(a, b) Internal fixation after clamshell osteotomy. (b) Blocking screw was used in distal tibia for proper positioning of rod which helped correction of deformity.

Clamshell osteotomy was performed as described by Russell et al. [1]. Patient was placed on radiolucent table, and radiolucent triangular bump was used to keep knee in 90 degree flexion position. Skin incision was made medial to patellar tendon of 3 cm extending proximal to tibial tuberosity. Plane was developed superficial to the fat layer taking care that joint is not exposed. Entry of guide wire was made just medial to lateral intercondylar eminence tibia proximal to tibial tuberosity and in line of the medullary canal under fluoroscopy. At this point, tip of guide wire was left just proximal to the fracture site. The proximal and distal site of the fracture was marked under the fluoroscopy and anterolateral skin incision was made just lateral to the shin of tibia. Skin and subcutaneous tissue were cut, muscles of anterior compartment were retracted laterally, and fracture site on tibia was exposed. The proximal and distal aspect of the extent of long oblique fracture was marked on tibia under fluoroscopy. The clamshell fragment of the bone was 8.5 cm long and several vertical drill holes were made on the anterior border of the tibia over clamshell fragment with a 2.5 mm drill bit. Drill holes extended through anterior and posterior cortex to create a path for the vertical osteotomy. This was followed by transverse osteotomies performed through the proximal and distal parts of bone under fluoroscopy control with oscillating saw followed by vertical osteotomy through the drill holes in the middle fragment with oscillating saw. The middle fragment opened up like a clamshell. Then, 1 cm of fibula was excised through a separate lateral incision over fracture site to allow good collapse of the osteotomy and was used as bone graft. Tibia was properly aligned and “blocking screw” was placed laterally on the distal fragment to maintain corrected varus position of tibia (Figures 3(a) and 3(b)). Guide wire was passed from proximal to the distal fragment and fascia and subcutaneous tissue were closed over the osteotomy site to preserve the reamings. Tibial canal was reamed with 9–13 reamers and no 12 tibial nail (Biomet Versa) was inserted. Nail was locked with two proximal and two distal locking screws (Figures 3(a) and 3(b)).

**Figure 4:**
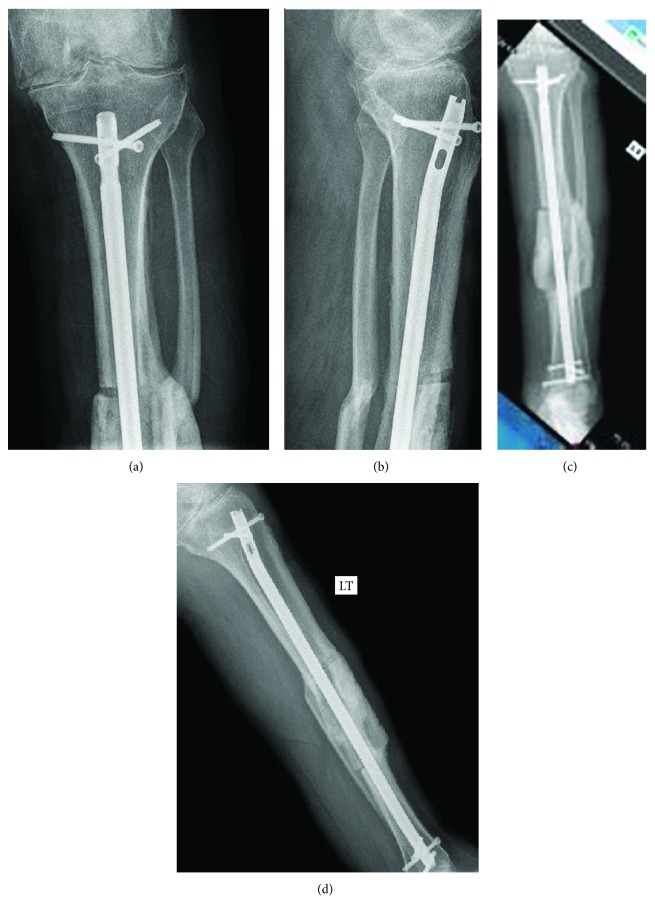
(a–d) Postoperatively showing good healing at fracture site at 6 months.

**Figure 5:**
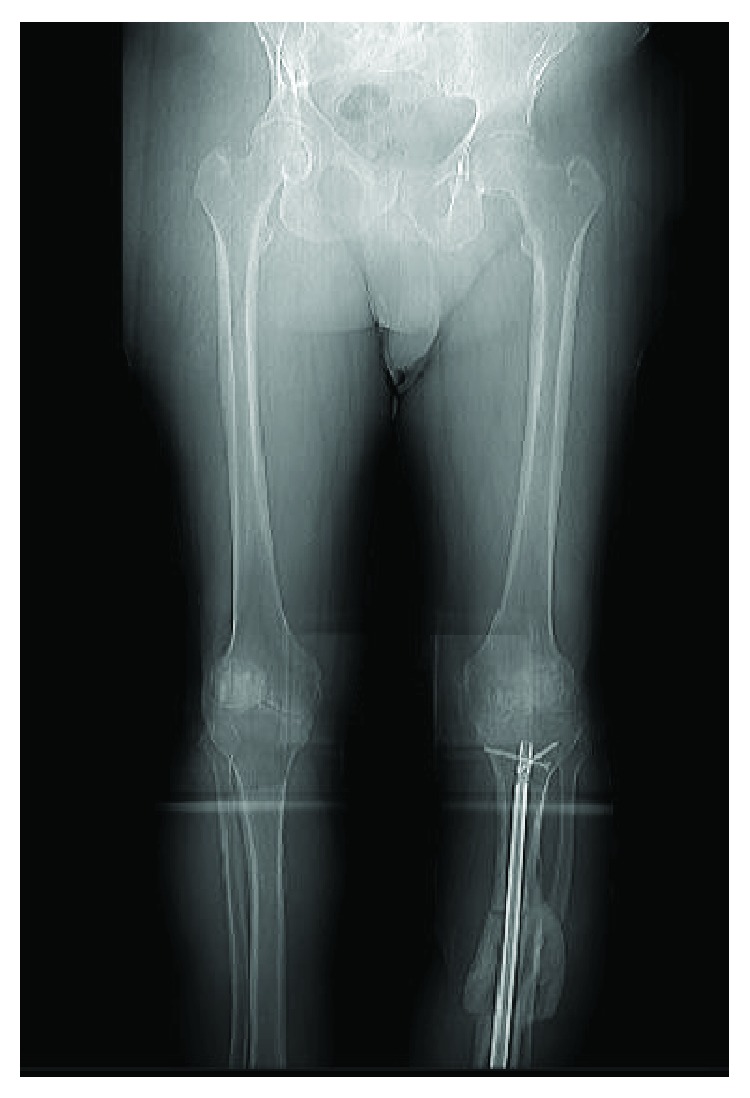


There are several studies which have concluded that calcium and vitamin D levels affect healing of the fracture [2, 3]. So calcium and vitamin D levels were monitored and corrected as needed after surgery. Blood sugar was controlled postoperatively. Radiologic healing of fracture was confirmed by three cortices healing on X-ray at 6 months (Figures 4(a)–4(d)). CT scan of the left knee and tibia was done (Figure 5) to confirm union and preoperative planning for custom-made cutting blocks to avoid intramedullary templating or instrumentation during total knee replacement.

### 3.1. Planning and Surgical Procedure for Total Knee Replacement

Plan was made for extramedullary referencing for both tibia and femur. Custom-made cutting blocks were prepared prior to the surgery (Zimmer Biomet, Signature system; Warsaw, IN, USA) based on CT scan. The femoral valgus angle was set to 4.5 degrees and tibial varus-valgus angle was set to 0 degrees. The posterior slope on tibia was set to 3 degrees and tibial resection below lateral low point was 8 mm while below medial low point was 2 mm.

During surgery, two proximal screws from tibia nail were taken out using a fluoroscope. Plan was made for removal of the tibial nail if needed. A medial knee parapatellar approach for total knee replacement was made and distal femur and tibia cuts were made using custom made jig (Zimmer Biomet Signature). Distal femur, patella, and proximal tibial preparation was done. There was no interference of nail during tibial canal reaming and impaction of wings of tibial component. Remaining surgery was performed in routine manner.

**Figure 6:**
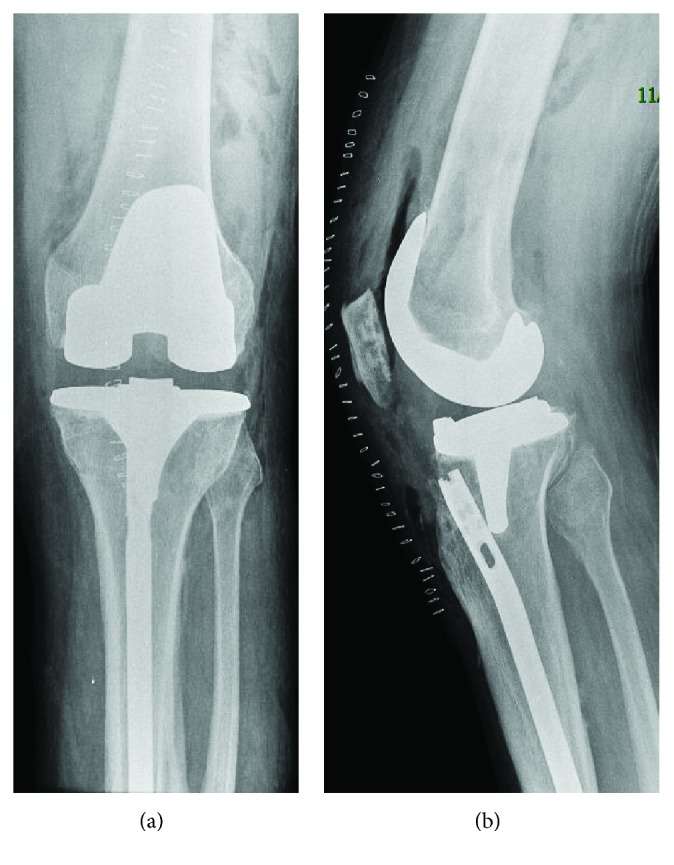
Total knee replacement after removing proximal tibial nail screws. Removal of IM nail was not indicated.

Postoperative X-rays showed good alignment (Figure 6). Mechanical axis of lower extremity was restored to 3 degrees with vertical axis of the body. Patient was started physical therapy and weight bearing operated extremity on same day. Patient regained 0–95 degrees of knee movement at 4 weeks and had 110 degrees movement at 6 months.

## 4. Discussion

Clamshell osteotomy reported by Russell et al. [1] was preliminary experience at three institutions in USA. Two more case report of clamshell osteotomy have been reported in the literature [4, 5]. Wilson et al. reported a case of total knee arthroplasty after correction of extra-articular femoral deformity caused by malunited femoral shaft fracture. To our knowledge, this is the first case of total knee arthroplasty after correction of extra-articular deformity in tibia using clamshell osteotomy.

Deformities in tibia which included varus, anterior angulation, translation, rotation, and shortening were corrected using this technique. The placement of blocking screw lateral to the nail helped in preventing distal fragment going into varus. Russell et al. [1] have suggested avoiding correction of shortening more than 5 cm in femur and 3 cm in tibia. Shortening in our patient was 1.5 cm and we did not have any difficulty in lengthening 1.5 cm. There were no clinical findings of excessive neurovascular stretching postoperatively. The fibular segment excised was used as bone graft and we did not use any allograft or synthetic osteoconductive materials. We also considered regarding adequate soft tissue coverage and early weight bearing as described by Russell et al. [1]. Our patient had malunited tibial fracture for 20 years and skin coverage over deformity site was adequate. Intramedullary nail helped in early weight bearing as it is load sharing device [6].

Fracture malunion, previous failed osteotomies, epiphyseal injury, osteomyelitis, metabolic bone disease involving bone, and Paget's disease are the major causes for extra articular deformity in long bones. Deformities are considered extra-articular in the knee joint when proximal to femoral epicondyles or distal to the tip of fibula [7]. Alignment in case of extra articular deformity could be corrected by osteotomy at deformity site, osteotomy away from the deformity, or intraarticular correction through cuts and positioning of prosthesis.

Sabharwal et al. [8] and Kocaoglu et al. [9] have described a method of combined external fixation with internal fixation for the correction of deformity and have favored it in deformities resulting from metabolic bone diseases. It adds to additional stability. However, pin site infection is common after external fixation and is reported between 2 and 30% [10]. Osteomyelitis is one of the fatal consequence of pin site infection after external fixation and can delay TKA [11]. Surgical time is also increased when both internal fixation and external fixation procedures are combined [12]. We had considered combining external fixation in the event of implant loosening, delayed union, or nonunion followed by removal of external fixation after healing of fracture and TKA after healing of pin site wounds.

Rajgopal et al. have reported correction of extra-articular deformity of 18 degrees in coronal plane and 15 degrees in sagittal plane for femur and 24 degrees in coronal plane in tibia with intra-articular resection [13]. They suggested that a deformity greater than 24 degrees in tibia or associated deformity in sagittal plane and rotational plane needs extra-articular correction either during total knee arthroplasty or as a stage procedure prior to surgery. Wolff et al. has shown that correction of large extra-articular deformities with large wedge resection of femur or tibia between attachments of collateral ligaments of knee will result in asymmetrical ligament length and complex instabilities around the knee [14]. Our patient had deformity in coronal, sagittal, and rotational planes and so the chances of instability were very high if deformity was just corrected with intra-articular resection of tibia. Moreover, patient was diabetic with high BMI and so staged procedures for the management of osteoarthritis knee were planned.

The use of intramedullary guide for wedge resection of tibia was not possible due to presence of nail. The patient had long-standing rotational malalignment and previous surgery in tibia. This increased chances of rotational malalignment using extramedullary tibial guide. MRI-guided custom jig preparation was not possible due to presence of hardware around the knee. So we got CT-guided custom jig prepared for total knee replacement.

## 5. Conclusion

Clamshell osteotomy is an excellent procedure to correct an extramedullary deformity in the tibia in coronal, sagittal, and rotational plane prior to total knee arthroplasty. It reduces the amount of deformity correction needed during total knee arthroplasty. Tibial nail does not require removal and TKA can be done as soon as we can see clinical and radiologic evidence of healing at osteotomy site with early return of patient to normal activities of daily living.
